# Views on GWAS statistical analysis

**DOI:** 10.6026/97320630016393

**Published:** 2020-05-31

**Authors:** Xiaowen Cao, Li Xing, Hua He, Xuekui Zhang

**Affiliations:** 1 Department of Mathematics, Hebei University of Technology, Tianjin, China; 2 Department of Mathematics and Statistics, University of Saskatchewan, Saskatoon, SK, Canada; 3 Department of Mathematics and Statistics, University of Victoria, BC, Canada

**Keywords:** Genome-Wide Association Studies, Single Nucleotide Polymorphisms, Statistical power, Multiple Testing Adjustment, Linkage Disequilibrium, Supervised Learning, Unsupervised Learning

## Abstract

Genome-wide association study (GWAS) is a popular approach to investigate relationships between genetic information and diseases. A number of associations are tested in a study and the results are often corrected using multiple adjustment methods. It is observed that GWAS studies suffer adequate statistical power for reliability. Hence, we document known models for reliability assessment using improved statistical power in GWAS analysis.

## Background

Genome-wide association study (GWAS) is a popular approach to investigate associations between genetic information and diseases [1]. Known literature on GWAS is large (213,610 records) as of June 2020 in PubMed [2]. There were about 5687 GWAS studies (September 2018) submitted to the NHGRI-EBI GWAS Catalog [3]. This catalog documented 175,870 associations from 4439 studies (February 2020) [4]. The observed data contain the genotype of hundreds of thousands of Single Nucleotide Polymorphisms (SNPs). We test the association between disease outcomes for each SNP one by one. Hence, multiple testing adjustments are critical to control false positive results when many tests are conducted in a single study [5]. Typical multiple testing procedures include the Bonferroni correction with the GWAS threshold of 5x10e-8 towards controlling the False Discovery Rate (FDR). A large proportion of the GWAS studies suffer lack of adequate statistical power due to large data dimensionality. Therefore, it is of interest to review approaches to address 'lack of statistical power' in GWAS analysis with large sample size.

## Large GWAS dataset:

Data size is critical in GWAS analysis. The per-sample cost of genomic studies reduced substantially at a speed much faster than Moore's law [6] due to the advancement in high-throughput technologies such as the next generational sequencing [32]. This makes it possible to conduct genomic studies using large sample size. A number of such studies have been completed. For example, the UK Biobank Data [7] gleaned genomic data with related information from over 500,000 volunteers. Large proportion of meta-data (derived data) using the UK Biobank Data is available. The Electronic Medical Records and Genomics (eMERGE) network [8] is a National Human Genome Research Institute–funded consortium engaged in the development of methods and best practices to connect genomic data to electronic medical records. This study collected data for 39 million SNPs from 100,000 participants.

A number of software tools are available to analyze GWAS data. Plink [9] and snpStats [10] are the most popular tools. These tools implement SNP-wise (one-by-one) testing followed by multiple testing adjustments. Plink is a widely used toolset for GWAS. The basic association test is for a disease trait and is based on comparing allele frequencies of SNPs between cases and controls. Alternative tests are also implemented in Plink. These include the Cochran-Armitage trend test, Fisher's exact test, different genetic models (dominant, recessive and general), tests for stratified samples and a test for a quantitative trait. Multiple testing adjustments to control false positive probability are conducted in these tests for every SNP. Popular adjustment options (such as Bonferroni, Sidak, and FDR) are also implemented in Plink.

The snpStats is an R package in Bioconductor for GWAS. The snpStats can handle both quantitative and qualitative phenotypes. It can carry out single SNP tests adjusted for potential perplexing by quantitative and qualitative covariates. Tests having several SNPs taken together as 'tags' are also supported in these analyses. The snpStats package offers options for quality control using Hardy Weinberg equilibrium tests and filtering SNPs using minor allele frequencies. Similar to Plink, snpStats also offer popular multiple testing adjustments options. Plink and snpStats are freely downloaded and the detailed instructions of the various functions in the programs can be found in the respective user manuals.

## Statistical models in GWAS:

### The linkage disequilibrium:

Improving the statistical power using large sample size is not suitable for all genomic studies. This is because of majority of studies have enough samples to generate huge data as required. Therefore, it is desired to improve the study power using novel statistical analysis methods. The development of novel methods is gaining momentum over the last decades. Standard analysis method tests each SNP separately, but the SNPs are correlated with each other. The relationship between SNPs is called Linkage Disequilibrium (LD), which provides information for other SNPs that are in linkage with each other [11]. Most novel statistical models are developed by properly incorporating the LD relationship among SNPs to allow the tests use information from each other.

The number of parameters in the LD matrix is n(n-1)/2, where n is the number of SNPs being investigated. Hence, it is not realistic to obtain a precise estimation of LD matrix using moderate amount of samples in a genomic study. Hence, all reliable models incorporate LD information without clarity clearly. These do not use estimated LD matrix as model parameters as described below.

### Supervised learning approaches:

The genomic information is the input data and the disease is the outcome in the association study using a supervised machine-learning model. There are various complex statistical models developed to improve the statistical power for SNP detection. We consider all of these methods as supervised learning methods, which comprises of SNP-set analysis [12， 13,14,15], Penalized regression approach [16, 17,18] and Bayesian hierarchical regression models [19,20, 21,22].

### Unsupervised learning approaches:

Model-based clustering is an unsupervised machine learning method, which can be used to group SNPs. The SNPs in the same group have similar relationship to the outcome, and could borrow information from each other in the GWAS analysis. A recent method proposed a one-step model. This simultaneously clusters SNP and detects significant SNP with FDR control [23].

The patterns of clusters are specified by the difference in minor allele frequencies of SNPs between cases and controls. Thus, the pattern is enforced with a special prior distribution. This model-based clustering have shown more precise controls of FDR and higher statistical power in both simulation studies and real data analysis [23]. The limitation is that it can only handle case-control association studies.

### Data splitting approach:

The other approach is based on data splitting strategy. The data can be randomly split into a screening set and a testing set. We use the screening set to remove the majority of SNPs with weak signals; and then investigate the retained SNPs in the testing set. The test sets only consider a very small subset of SNPs. This leads to fewer penalties in the multiple test adjustment on testing set. So, this approach is much more powerful than analyzing the original data with all SNPs. The results of this type of analysis can be heavily affected by which samples are split into the testing set. We use resampling approaches to analyze multiple copies of the data with different random splits to remove unwanted 'split' effect [24,25]. These methods are not popular since they have multiple critical disadvantages. First, multiple testing adjustment method is not available for controlling false positives in these methods. Second, such methods involve multiple tuning parameters whose values have to be selected in an ad-hoc manner.

## Conclusion:

GWAS is a popular method to study genome relationship with diseases and their linked phenotypes. The adjustment of multiple testing is critical to reduce false positives in these studies. It is well realized that many GWAS studies suffer statistical power in SNP discovery due to high dimensional description of the problem at hand. Hence, it is of interest to document known information on large studies and known statistical analysis models that are pertinent to GWAS. Thus, application of novel statistical analysis models on large datasets using high performance computing (HPC) infrastructure is highly recommended.

## References

[1] Manolio TA (2010). N Engl J Med.

[02] https://pubmed.ncbi.nlm.nih.gov/?term=GWAS.

[3] Buniello A (2019). Nucleic Acids Res.

[4] https://www.ebi.ac.uk/gwas..

[5] Chen SY (2017). J Thorac Dis.

[6] https://www.genome.gov/about-genomics/factsheets/Sequencing-Human-Genome-cost..

[7] Elliot LT (2018). Nature.

[8] Gottesman O (2013). Genet Med.

[9] Chang CC (2015). GigaScience.

[10] Clayton D, Leung HT (2007). Hum Hered.

[11] Slatkin M (2008). Nat Rev Genet.

[12] Cologne J (2018). BMC Genomics.

[13] Dai H (2013). PLoS One.

[14] Lu ZH (2015). Genet Epidemiol.

[15] Wu MC (2010). Am J Hum Genet.

[16] Kang HM (2010). Nat Genet.

[17] Yu J (2006). Nat Genet.

[18] Zhou X, Stephens M (2012). Nat Genet.

[19] Chen C (2017). Genetics.

[20] Fernando RL, Garrick D (2013). Methods Mol Biol.

[21] Sanyal N (2019). Bioinformatics.

[22] Wang Q (2016). J Anim Breed Genet.

[23] Xu Y (2019). Sci Rep.

[24] Kang G (2015). J Hum Genet.

[25] Yang JJ (2012). Blood.

[26] Bansal V (2010). Nat Rev Genet.

[27] Magrangeas F (2016). Clin Cancer Res.

[28] Xu Z, Taylor JA (2009). Nucleic Acids Res.

[29] Eskin E (2008). Genome Res.

[30] Darnell G (2012). Bioinformatics.

[31] Goode EL (2011). Linkage Disequilibrium. In: Schwab M. (eds)Encyclopedia of Cancer..

[32] Xu Feng (2012). Nat Commun.

